# Controlling resistive switching behavior in the solution processed SiO_2-x_ device by the insertion of TiO_2_ nanoparticles

**DOI:** 10.1038/s41598-022-12476-y

**Published:** 2022-05-19

**Authors:** Sera Kwon, Min-Jung Kim, Dong-Hyeok Lim, Kwangsik Jeong, Kwun-Bum Chung

**Affiliations:** 1 Division of Physics and Semiconductor Science, Dongguk University, Seoul, 04620 Republic of Korea; 2 Department of Materials Science and Engineering, UNIST, Ulsan, 44919 Republic of Korea

**Keywords:** Applied physics, Condensed-matter physics, Electronics, photonics and device physics, Materials for devices, Nanoscale materials, Nanoscale devices, Nanoscale materials

## Abstract

The resistive switching behavior of the solution processed SiO_x_ device was investigated by inserting TiO_2_ nanoparticles (NPs). Compared to the pristine SiO_x_ device, the TiO_2_ NPs inserted SiO_x_ (SiO_x_@TiO_2_ NPs) device achieves outstanding switching characteristics, namely a higher ratio of SET/RESET, lower operating voltages, improved cycle-to-cycle variability, faster switching speed, and multiple-RESET states. Density functional theory calculation (DFT) and circuit breaker simulation (CB) were used to detail the origin of the outstanding switching characteristic of the SiO_x_@TiO_2_ NPs. The improvement in resistive switching is mainly based on the difference in formation/rupture of the conductive path in the SiO_2_ and SiO_2_@TiO_2_ NPs devices. In particular, the reduction of resistance and lower switching voltage of TiO_2_ NPs control the formation and rupture of the conductive path to achieve more abrupt switching between SET/RESET with higher on/off ratio. This method of combined DFT calculation and CB offers a promising approach for high-performance non-volatile memory applications.

## Introduction

Ongoing research on the resistive random-access memory (ReRAM) has allowed outstanding performance that includes non-volatility, fast switching speed, and low power consumption^1–7^. Recently, ReRAM has attracted much interest as a promising candidate for next-generation non-volatile memory, and displays suitability for applications such as neuromorphic electronics^8–11^. The binary-metal oxide resistance switching characteristics have been extensively studied for an active layer, including TiO_2_, Ta_2_O_5_, ZnO, SiO_2_, and HfO_2_, due to their simple compositions with adjustable stoichiometry^2,12–15^. Among them, the device with amorphous form of SiO_x_ that is constructed as an active layer sandwiched between electrodes shows remarkable resistive switching behavior and transparency^16–19^. SiO_x_ is known to have relatively low variability and outstanding stability, which properties lead to a high resistance window for sufficient read margin between high resistance state (HRS) and low resistance state (LRS)^11,20,21^. Meanwhile, the SiO_x_-based resistive switching devices have achieved superior switching characteristics and reliability by using several device architectures that include the nanopillar-structured SiO_x_ fabricated with nanosphere lithography, exposed sidewall etched into the SiO_2_ layer, and nanoporous SiO_x_-based memory structures^14,22,23^. In addition, the modulation of resistive switching properties is obtained by combination with an additional layer or insertion of structures into the SiO_x_-based matrix^16,24–26^. As known, most research efforts are mainly based on firms that fabricate using atomic layer deposition (ALD), plasma-enhanced chemical vapor deposition (PECVD), electron-beam evaporation, and magnetron sputtering, which need vacuum techniques that are complicated and expensive^21,27–29^. Among various preparation methods as substitute for vacuum techniques, the solution process has shown superiority in its facile process, cost-effectiveness, applicability to various substrates, and adaptability to combination with several compositions or structures^30,31^. In addition, it is easy to insert the nanostructures into oxide matrix during the synthetic process, and this simple method is expected to control the characteristics of the switching performance.

Herein, we demonstrate a simple method using the insertion of TiO_2_ nanoparticles (NPs) to improve the resistive switching characteristics in terms of multi-level resistive switching performance of solution processed SiO_x_-based ReRAM. TiO_2_ NPs inserted SiO_x_ (denoted as SiO_x_@TiO_2_ NPs) shows superior resistive switching characteristics that include the higher ratio of SET/RESET states, lower SET/RESET voltages, and voltage controllable RESET state by applying external voltage, compared to the pristine SiO_x_. Furthermore, the resistive switching behavior is discussed by analyzing the electronic structure, as well as circuit-breaker simulation and theoretical calculation. These challenges are expected to make great contributions to the development of next-generation electronic devices.

## Results and discussion

### Structure of SiO_x_ based devices

**Figure 1:**
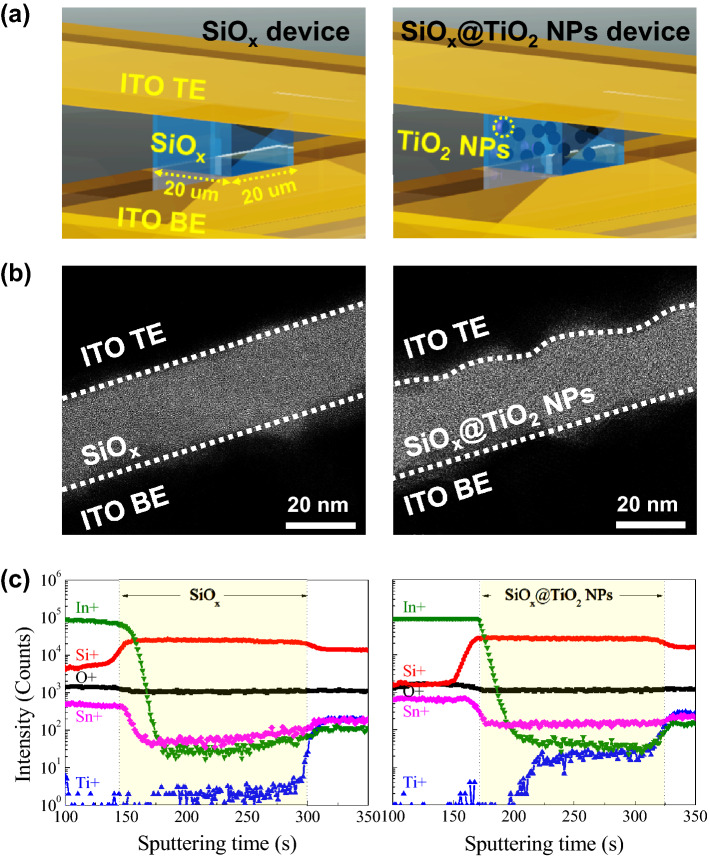
(**a**) Schematic structures of the cross-bar array architecture, (**b**) the cross-sectional TEM images, and (**c**) depth-profiling with ToF–SIMS of the SiO_x_ and SiO_x_@TiO_2_ NPs resistive switching devices.

Figure 1a schematically illustrates the cross-bar array architectures of the SiO_x_ and SiO_x_@TiO_2_ NPs resistive switching devices. And, to observe the cross-sectional information of both devices, TEM is measured as shown in Fig. 1b. The TEM image of the SiO_x_ device shows that the ITO/SiO_x_/ITO structure is sequentially stacked, and the interface of TE/SiO_x_ is clearly formed. In contrast, the SiO_x_@TiO_2_ NPs device shows the slightly rough interface of TE/SiO_x_@TiO_2_ NPs, which is related to the insertion of TiO_2_ NPs profoundly affecting the roughness of SiO_x_@TiO_2_ NPs. To examine the composition of the SiO_x_ and SiO_x_@TiO_2_ NPs device structures, ToF–SIMS was measured from top to bottom electrode during O ion sputtering with 2 keV. Figure 1c shows that the spectra of ToF–SIMS can be divided into three regions; the first region is only ITO (top electrode), the second region is the SiO_x_ or SiO_x_@TiO_2_ NPs layer, and the last region is ITO (bottom electrode). For the SiO_x_ switching device, Si^+^ is increased in the second region, while In^+^ and Sn^+^ are drastically decreased. O^+^ is continuously detected in all regions, because oxygen is included in all layers. Ti^+^ is not detected in the first and second regions. In the third region, Ti^+^ is found due to the glass substrate, thus it can be negligible^32^. In the case of the SiO_x_@TiO_2_ NPs device, the behaviors of Si^+^, In^+^, Sn^+^, and O^+^ are almost similar to those of the SiO_x_ device. However, a considerable quantity of Ti^+^ is detected in the second region, and we can recognize that the TiO_2_ NPs is well inserted into the SiO_x_ matrix.

### Analysis of the chemical bonding states

**Figure 2:**
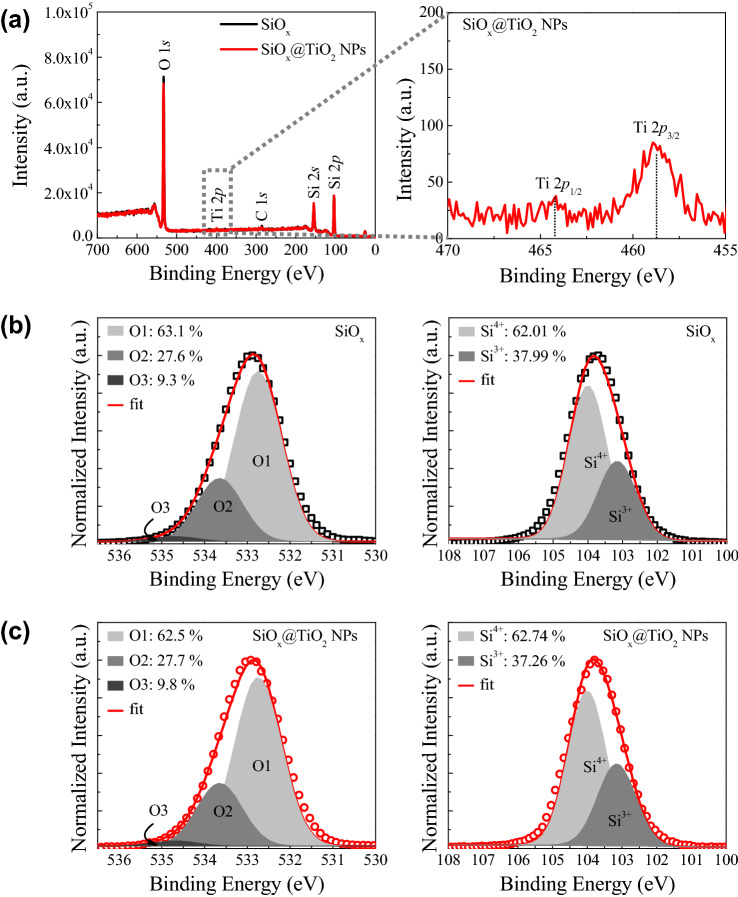
(**a**) XPS survey (left), and the enlargement of Ti 2*p* range (right), for the SiO_x_@TiO_2_ NPs. Core-level spectra of O 1* s* and Si 2*p* for the (**a**) SiO_x_, and (**b**) SiO_x_@TiO_2_ NPs.

Figure 2 shows the compositions and chemical bonding states of the SiO_x_ and SiO_x_@TiO_2_ NPs films by using XPS measurement. Both films are composed with O, Si, and a small amount of C, and Ti is included to ~ 1.5% in the SiO_x_@TiO_2_ NPs layer, as shown in Fig. 2a. To elucidate the chemical bonding states, the core-level spectra of Si 2*p* and O 1* s* were normalized, and deconvoluted into Gaussian peaks. In the case of O 1* s*, it is composed with three Gaussian peaks according to Si–O bonds (O1s), oxygen deficient states (O2), and hydroxyl groups (O3), as shown in Fig. 2b and c^26,33^. The prepared SiO_x_ and SiO_x_@TiO_2_ NPs films have a lot more O2 and O3 states than does the conventional SiO_2_ film. Generally, this is related to the solution processed SiO_x_ obtaining a large amount of defect states, such as oxygen vacancies or OH groups, which affects the stoichiometry of the SiO_x_ (x < 2)^26,28^. In the Si 2*p* spectra, the regular SiO_2_ (Si^4+^) and oxygen deficient SiO_2-x_ (Si^3+^) are indicated, as shown in Fig. 2b and c^34,35^. The prepared SiO_x_ films synthesized by solution process show higher composition of oxygen vacancies than the SiO_2_ synthesized by vacuum process (thermal oxidation or chemical vapor deposition). In general, the amount of oxygen vacancies is expected to change due to the difference in bond dissociation energy in the TiO_2_ NPs inserted SiO_x_ system^27,36^. However, in our system, the chemical bonding states are almost similar, due to the small amount of TiO_2_ NPs in SiO_x_ matrix. Therefore, the change of the chemical bonding states of SiO_x_ is imperceptible.

### Resistive switching characteristics

**Figure 3:**
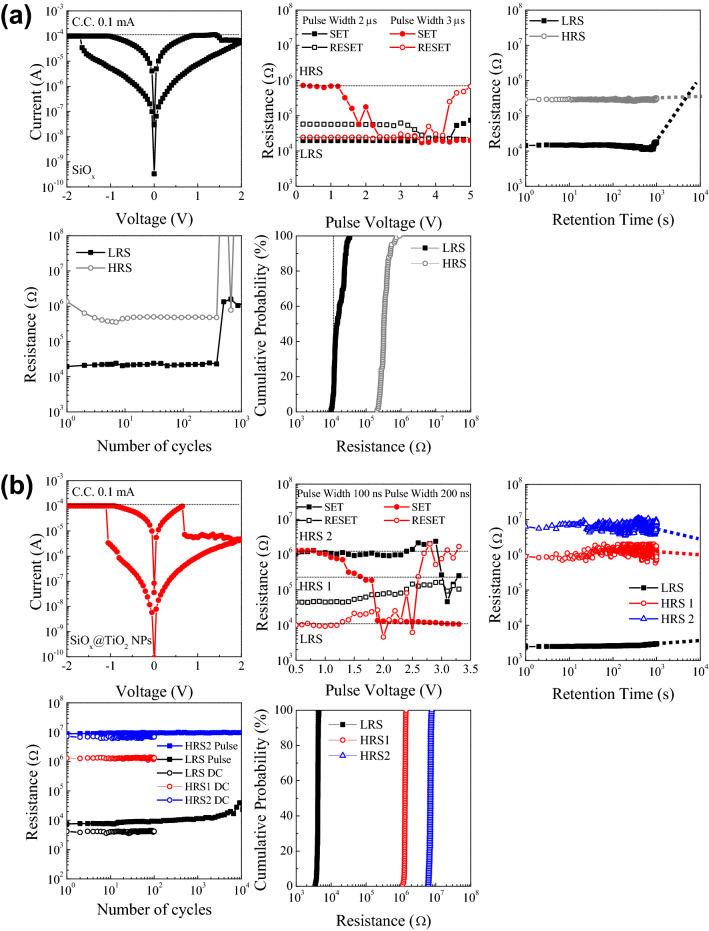
I–V characteristics, switching speed, retention (dotted lines indicate the expectation of lifetimes for the devices), endurance, and cycle-to-cycle variability of the (**a**) SiO_x_ and (**b**) SiO_x_@TiO_2_ NPs resistive switching devices.

Figure 3 shows the resistive switching performance of the SiO_x_ and SiO_x_@TiO_2_ NPs devices. First, the voltage is swept to transit from the pristine state to LRS with the compliance current of 0.1 mA. Both devices show bipolar resistive switching behavior with SET process, which is obtained by sweeping the negative voltage, while the RESET process is obtained by sweeping the positive voltage. These processes can be reversibly changed by controlling the voltage. In detail, the SiO_x_ device switches from HRS to LRS (SET process) at − 1.7 V, while the device switches continuously from LRS to HRS (RESET process) by applying voltage to 2.0 V. For the SiO_x_@TiO_2_ NPs device, the SET process occurs at − 1.1 V, which is smaller than the SET voltage of the SiO_x_ device. In addition, different from the SiO_x_ device, the RESET process is obtained twice over; the first RESET process appears at 0.7 V, then the second RESET process with slight resistance change is obtained while increasing the voltage to 2.0 V. The switching speed is obtained that the RSs change under the pulse width of 3 us in SiO_x_ device. In case of SiO_x_@TiO_2_ NPs device, the RSs transit with the pulse width of 200 ns, as shown in Fig. 3a and b. To evaluate the reliability of devices, the retention and endurance were conducted for SiO_x_ and SiO_x_@TiO_2_ NPs. The retention test was performed by probing each LRS and HRS for 10^3^ s at room temperature, and reading the current at 0.1 V. The SiO_x_ device maintains the LRS/HRS ratio of approximately 20 for 10^3^ s. For the SiO_x_@TiO_2_ NPs device, three well-defined RSs (LRS, HRS1, and HRS2) are maintained for 10^3^. Also, the dotted lines indicate the expectation of the lifetime of two devices. The SiO_x_ device shows the expectation of the lifetime almost 10^3^ s, otherwise the SiO_x_@TiO_2_ NPs device is expected to the lifetime above 10^4^ s. To examine the endurance performance, the SET/RESET cycling test was conducted for 10^2^ times, and the current level was recorded at 0.1 V. Both devices show a stable LRS/HRS ratio for 10^2^ cycles. In particular, the SiO_x_@TiO_2_ NPs exhibits the multiple RSs for 10^2^ cycles. Moreover, the endurance of SiO_x_@TiO_2_ NPs is evaluated for 10^4^ cycles under the pulse width of 200 ns. The device shows the stable operation during 10^4^ cycles. Long retention time and stable endurance indicate the high reliability of the solution processed SiO_x_-based resistive switching devices. The distribution of SET and RESET voltages are measured for 10^2^ times, and the current level was recorded at 0.1 V for examining the cycle-to-cycle variability of devices. Both devices show a stable LRS/HRS ratio for 10^2^ cycles. Especially, the SiO_x_@TiO_2_ NPs exhibits the multiple RSs for 10^2^ cycles, and the stable resistive switching occurs in the SiO_x_@TiO_2_ NPs device, compared to SiO_x_ device. The stable endurance, retention, cycle-to-cycle variability performance indicate the high reliability of the solution processed SiO_x_-based resistive switching devices. From the resistive switching characteristics, it is concluded that the SiO_x_@TiO_2_ NPs device can be expected to high performance and low-power non-volatile memory due to a lower operation voltage, higher on/off ratio, and fast switching speed^23^. Moreover, due to multi-level switching, the SiO_x_@TiO_2_ NPs device is also applicable to multi-level memory.

### Resistive switching mechanism

**Figure 4:**
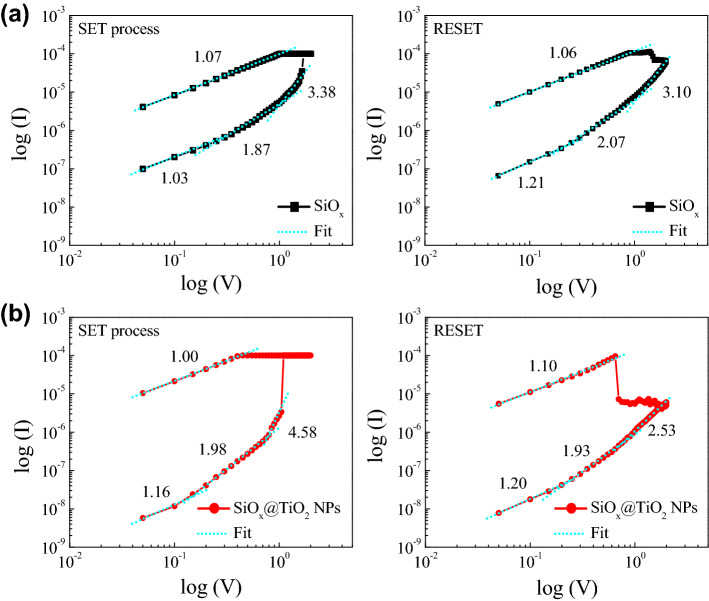
Log I–log V plot of the I–V curves of the (**a**) SiO_x_, and (**b**) SiO_x_@TiO_2_ NPs in the SET and RESET processes.

To discover the origin of the enhancement of performance in the SiO_x_@TiO_2_ NPs device, we clarified the resistive switching mechanism of the SiO_x_ and SiO_x_@TiO_2_ NPs devices. The I–V curves are re-plotted as log I–log V, as shown in Fig. 4a and b. In the SET process of the SiO_x_ resistive switching device, the I–V curve of the HRS exhibits trap-controlled space charge limited current (SCLC) conduction, which is composed of three parts: the Ohmic region (I ∝ V), the Child’s law region (I ∝ V^2^), and the steeply increasing region (I ∝ V^n^, n > 2)^37^. The oxygen vacancies in the SiO_x_ matrix serve as an electron trap, and form the conductive filament. Thus, the migration of oxygen vacancies is an important role in the deviation of slopes. In the high-voltage region, all traps are filled with electrons, and excessive electrons flow through the conduction band of SiO_x_ (achievement of the SET process). The I–V curve of the LRS shows a linear Ohmic behavior with a slope of 1.07. Likewise, the RESET process is also in good agreement with the trap-controlled SCLC mechanism in HRS. In the SiO_x_@TiO_2_ NPs, the resistive switching mechanism is similar to that of the SiO_x_ device, as shown in Fig. 4b. The electrons are transported according to traps, such as oxygen vacancies, into the SiO_x_ matrix, as well as TiO_2_ NPs, and with the application of high voltage, then flow into the conduction band of SiO_x_ and TiO_2_. This behavior is associated with the bulk-controlled mechanism, such as the conductive filament model based on oxygen vacancy. As a result, the resistive switching mechanism of the SiO_x_ and SiO_x_@TiO_2_ NPs devices based on the conductive path can be dominated by valence change memory. The traps are a key factor to form the conductive paths into oxide matrix.

### DFT calculations

**Figure 5:**
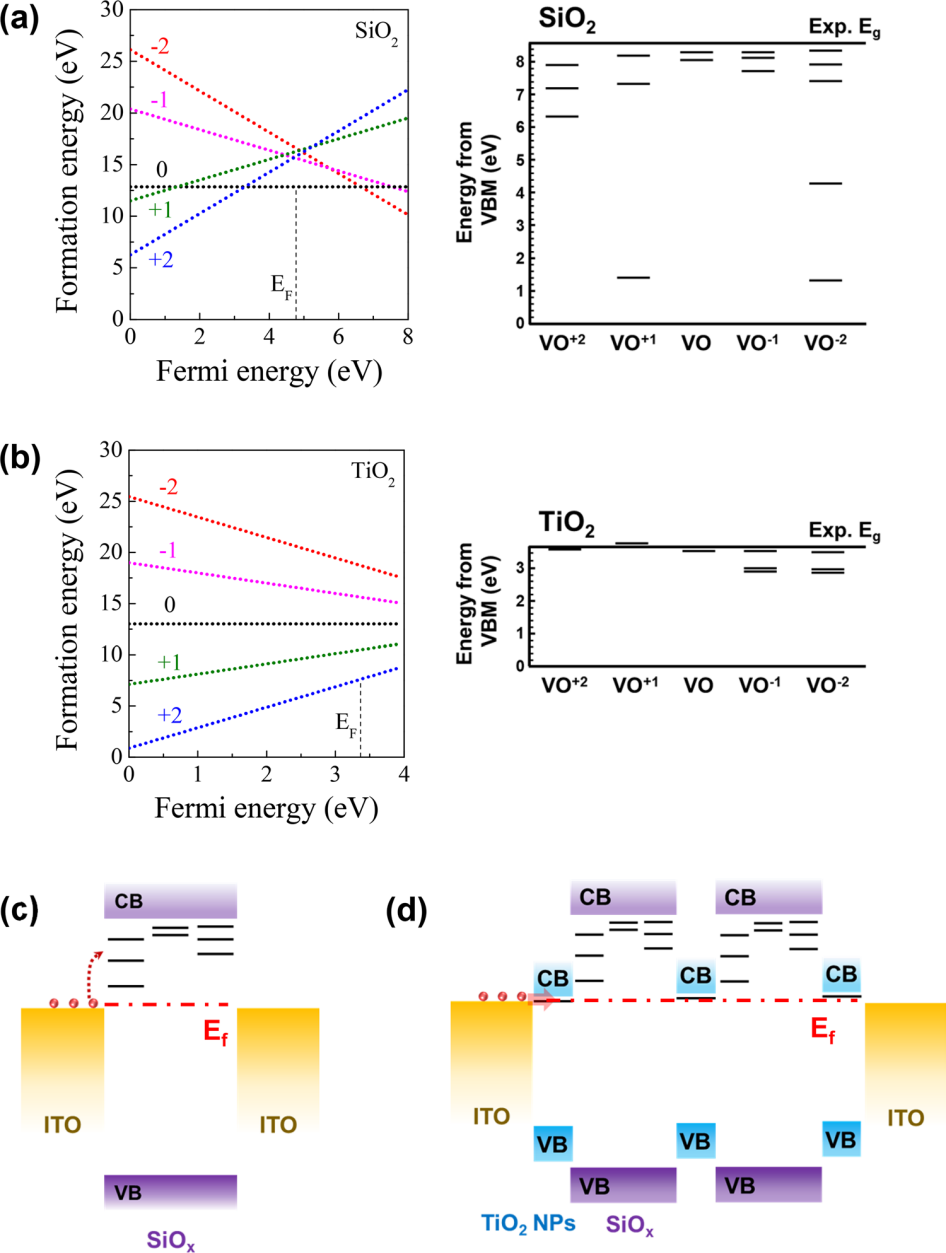
(**a**) Structural image of the SiO_x_, and (**b**) TiO_2-x_. (**c**) Defect states of the stable charge states of oxygen vacancies (V_O_) in SiO_x_, and (**d**) Fermi level-dependent formation energy of charge states of the V_O_ defects in SiO_x_. (**e**) Defect state of the stable charge of VO in the SiO_x_@TiO_2_ NPs, and (**f**) Fermi level-dependent formation energy of the charge states of VO defects in the SiO_x_@TiO_2_ NPs.

Furthermore, to predict the difference in conduction mechanism based on the oxygen vacancies (VO) defects of the SiO_x_ and SiO_x_@TiO_2_ NPs devices, DFT calculations for the defects in each oxide were conducted, as shown in Fig. 5. By considering stable crystal structure, α phase of SiO_2_ and the anatase phase of TiO_2_ were chosen for the calculations. Figure 5a and b show the Fermi level-dependent formation energies of the VO defects (VO^n^, where n = (− 2, − 1, 0, + 1, and + 2)) and the band structures containing the energy levels for each charging state of oxygen vacancies in SiO_2_ and TiO_2_, respectively. In addition, in the case of the VO in SiO_2_, the + 2 is the stable charging state for the energy range (0.0–3.2) eV, 0 is stable for the range (3.2–6.6) eV, and − 2 becomes stable above 6.6 eV. For the TiO_2_, the + 2 charging state is found to be the most stable state over the entire range of bandgap. Considering the Fermi level of SiO_2_ and TiO_2_, which is measured in the valance band spectrum of XPS, the most stable charging states of VO are 0 and + 2 for SiO_2_ and TiO_2_, respectively. Since the formation energy of VO in TiO_2_ is smaller than that of the VO in SiO_2_, VO is more easily generated in TiO_2_ than in SiO_2_. Moreover, since the e-field induced migration of VO can occur for charged states, VO in TiO_2_ that has 2 + charging states can migrate with smaller e-field.

Figure 5c and d show the schematic energy band diagrams of the SiO_x_ and SiO_x_@TiO_2_ NPs devices from the results of energy levels for stable charging states. Band alignments are estimated based on the valance band spectrum in XPS. For both the SiO_2_ and TiO_2_ systems, VO generates defect states in the bandgap, thus the resistance change of the RERAM device can occur through the generation (SET) and curing (RESET) of VO in oxide. However, considering the band alignment, the activation energy from the Fermi level to defect states is smaller in TiO_2_ than in SiO_2_. In addition, the barrier for carrier injection from ITO electrode is lower in TiO_2_ with VO than in SiO_2_ with VO. Therefore, the resistance of LRS in TiO_2_ is lower than the resistance of LRS in SiO_2_.

### CB simulations

**Figure 6:**
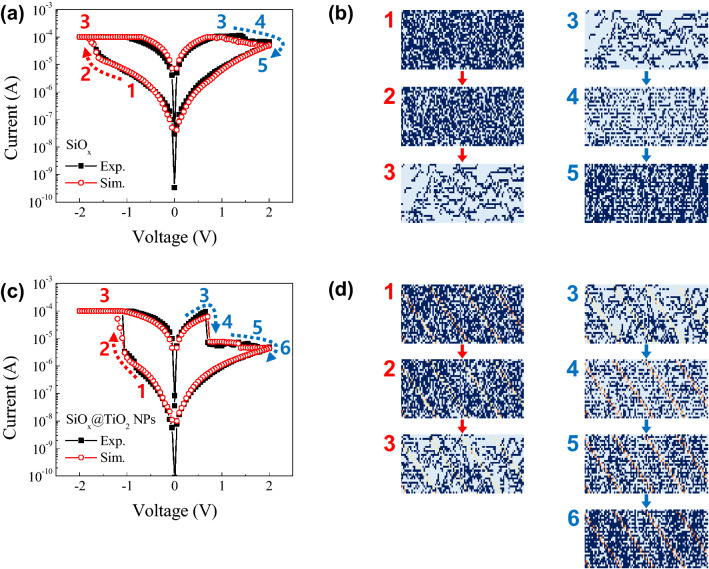
(**a**) and (**c**) Comparison of the experimental and simulated curves, and (**b**) and (**d**) maps of the circuit breakers with applying the voltages to the SiO_x_ and SiO_x_@TiO_2_ NPs resistive switching devices.

To understand the formation/rupture of the conductive path based on the oxygen vacancies for the SiO_x_ and SiO_x_@TiO_2_ NPs devices, Fig. 6 shows the stochastic circuit breaker (CB) simulation that was conducted. The simulation method was benchmarked to previous research, as reported by Brivio et al.^38^ In the SiO_x_ resistive switching device, both the experimental and simulated results show good agreement, which is also exhibited by the bipolar resistive switching behavior. The relative relations between parameters for SiO_2_ and TiO_2_ in CBs were determined based on DFT calculation. Table 1 tabulates the parameters. Figure 6b shows that the resistances of CBs are initialized with two values of R_high,S_ and R_low,S_ to simulate the insulating oxide and conducting oxide layer, such as SiO_2_ and oxygen-deficient SiO_2-x_, respectively. To emulate the oxygen-deficient SiO_x_ matrix, most of the CBs are initialized with R_high,S_, while the rest of the CBs are initialized with R_low,S_ in the initial state. In this case, the ratio of R_high,S_:R_low,S_ is set to approximately 6:4, and the switching probabilities of R_high,S_ and R_low,S_ depend on the electric field and temperature by Joule heating. On applying the negative volage, a few of the CBs are changed from R_high,S_ to R_low,S_ in sequence from #1 to #2 in Fig. 6b. Then, the voltage is applied above the SET voltage (> − 1.7 V), almost all CBs abruptly transform to R_low,S_, and the device achieves the SET process, as shown in #3 of Fig. 6b. In contrast, when the positive voltage is swept to the RESET voltage, R_high,S_ is continuously increased, then the CB network finally reaches the RESET process (according to the blue arrows in sequence from #3 to #5 in Fig. 6b). This cycle of SET/RESET is reversibly obtained on sweeping the voltage. Likewise, the experimental I–V curve of the SiO_x_@TiO_2_ NPs device agrees well with the simulated result, as shown in Fig. 6c. In Fig. 6 d, the maps of CB network are composed with four values of CBs of R_low,S_, R_high,S_, R_low,T_, and R_high,T_, which indicate SiO_2-x_, SiO_2_, TiO_2-x_, and TiO_2_, respectively. Initially, almost all CBs with R_high,S_ and R_low,S_ are randomly allocated in places. Based on the XPS analysis, the ratio of oxygen deficient R_low,S_ is equalized to that of the SiO_x_ device. The R_high,T_ and R_low,T_ of TiO_2_ NPs are also randomly distributed with the proportion of about 5%, to mimic the TiO_2_ NPs inserted SiO_x_ matrix. Similarly, the initial ratio of R_high,S_:R_low,S_ and R_high,T_:R_low,T_ is established to be about 6:4, respectively, as shown in Fig. 6d. On increasing the negative voltage, the CBs related to TiO_2_ NPs are more rapidly transited from R_high,T_ to R_low,T_ than those of SiO_2_ under the applied voltage. And, when further voltage is applied to the SiO_x_@TiO_2_ NPs device, the CBs related to SiO_x_ are also changed from R_high,S_ to R_low,S_, and the SET process is achieved according to the red arrows (in sequence from #1 to #3) in Fig. 6d. This is related to the TiO_2_ NPs assisting the construction of the conductive path in SiO_x_@TiO_2_ NPs, and causes lower SET voltages than that of the pristine SiO_x_ device. Under the positive voltage sweeps, the CBs of TiO_2_ NPs are rapidly changed from R_low,T_ to R_high,T_, while the CBs of SiO_x_ are slightly transited. Also, the first-RESET process can be achieved in sequence from #3 to #4, as shown in Fig. 6d. On further increasing the positive voltage, the R_high,S_ is increased, then the RS gradually reaches second-HRS (in sequence from #4 to #6 in Fig. 6d). The 2-step RESET processes can be obtained by controlling the RESET voltages.

**Table 1:** The parameters for CB simulations.

Parameters	SiO_x_ device	SiO_x_@TiO_2_ NPs device
M	30	30
N	90	90
R_low,S_ [kohm]	20	20
R_high,S_ [kohm]	9800	9800
R_low,T_ [kohm]	N/A	6
R_high,T_ [kohm]	N/A	8000
Initial ratio of fR_low,S_: R_high,S_	35:65	35:65
Initial ratio of R_low,T_: R_high,T_	N/A	36:64
I_C.C._ [mA]	0.1	0.1
Simulated V_SET_ [V]	− 1.7	− 1.1
Simulated V_RESET_ [V]	2.0	First-RESET: 0.7 Second-RESET: 2.0

In this study, the simulation method, which is simply expressed by the formation/rupture of conductive path with the stochastic CB model in the case of oxygen-deficient oxide matrix and nanoparticle-inserted oxide resistive memory devices, enables lower computational load for each CB network simulation than the conventional simulation methods. From the CB simulation, the conductive path based on the oxygen vacancies is stochastically examined under sweeping the external voltage. The difference in the switching of the SiO_x_ and SiO_x_@TiO_2_ NPs devices, such as the multiple-RESET, lowering switching voltage, and increase of on/off ratio, can be obtained by inserting the TiO_2_ NPs.

In our case, the SET process is abrupt by applying the negative voltages, which is related to the electric field inducing the defect migration, and then causing an increase of the current. In contrast, the gradual RESET process is due to the conductive filament being gradually ruptured when the positive voltage is applied to the devices^11,39^. Also, the improvement of the LRS/HRS ratio is noteworthy, as shown in Fig. 3a and b. This can be correlated to the environment for conductive filament growth inside the RS layer being changed due to the insertion of TiO_2_ NPs^40^. By applying the positive voltage, the conductive filament can easily rupture due to the existence of TiO_2_ NPs inside the SiO_x_ matrix, and carriers have difficulty in flowing inside the RS layer. Therefore, the current level of HRS for the SiO_x_@TiO_2_ NPs is lower than that of the SiO_x_ device.

## Conclusion

We demonstrate the improvement of the resistive switching behavior in the solution processed SiO_x_ device by the insertion of TiO_2_ NPs, which is structured with ITO/SiO_x_@TiO_2_ NPs/ITO on glass substrate. The SiO_x_@TiO_2_ NPs resistive switching device exhibits stable bipolar resistive switching behavior. Also, outstanding switching characteristics, such as the higher ratio of SET/RESET, lower SET/RESET voltages, improved cycle-to-cycle variability, faster switching speed, and controllable multiple-RSs (LRS, HRS1, and HRS2) by applying voltages, can be obtained, in comparison to the pristine SiO_x_ device. Based on stochastic circuit breaker simulation, we can conclude that the enhancement in switching performance in the SiO_x_@TiO_2_ NPs originates from the difference in the formation and rupture of conductive filament by the inserted TiO_2_ NPs.

## Methods

### Synthesis of solutions

SiO_x_ solution was synthesized by the sol–gel polymerization of silicon alkoxides. First, ethanol (C_2_H_5_OH, Aldrich, 99.9%) and deionized (DI) water were thoroughly stirred. A few minutes later, tetraethyl orthosilicate (TEOS, Si(OC_2_H_5_)_4_, Aldrich) was added as starting material. After that, hydrochloric acid (HCl, Merck, 37%) was added dropwise to the solution for 4 h, and then 0.1 M of sodium hydroxide (NaOH, Merck) was added for 16 h^41,42^. During the synthetic process, the solution was vigorously stirred at 500 rpm. After synthesis, the colorless and transparent SiO_x_ solution finally resulted.

### Fabrication of the SiO_x_ based devices

To fabricate the SiO_x_-based resistive switching memory device with cross-bar array architecture, the lift-off process was conducted on glass substrate^26^. The SiO_x_ solution diluted with ethanol was dropped on the patterned bottom electrode (BE), and spin-coated for deposition of 50 nm thick SiO_x_ at 5000 rpm for 60 s. In the case of SiO_x_@TiO_2_ NPs film, SiO_x_ solution and TiO_2_ NPs dispersed solution were mixed in the ratio of 1:7, and then spin-coated at the same condition. After that, the SiO_x_ and SiO_x_@TiO_2_ NPs films were dried at 80 °C for 20 min in oven, and then annealed using furnace at 450 °C for 1 h. The top electrode (TE) was also formed by using the lift-off process, and the cross-bar array ReRAM architecture was finally obtained with active device of 20 µm × 20 µm. During XPS measurement, Ar ion sputtering was conducted at 500 V for 10 s to eliminate carbon contamination on the surface.

### Analysis

The cross-sectional specimens were prepared with a focused ion beam (FIB, FEI Helios 650) system, and field effect transmission electron microscopy (TEM, JEOL Ltd. JEM-F200) was obtained. The composition was examined by time-of-flight secondary-ion mass spectrometry (ToF–SIMS, IONTOF, TOFSIMS5) with 30 keV of Bi ion with spot size of 35 µm × 35 µm, and the depth profile was obtained with 2 keV of O ion sputtering.

To investigate the composition and chemical bonding state of the SiO_x_ and SiO_x_@TiO_2_ NPs, X-ray photoelectron spectroscopy (XPS, ESCA Versaprobe II) was conducted by monochromatic X-ray radiation at energy *hv* = 1486.7 eV (Al Kα source) with pass energy of 29.5 eV. The resistive switching behavior was observed using current–voltage (I–V), which was measured by semiconductor analyzer (Keithley-4200). To contact the bottom electrode, the upper SiO_x_ layer was lightly scraped off using a thin tip because the bottom electrode was completely covered with SiO_x_ layer. During I–V measurement, the voltage was applied to TE, and BE was grounded. The electronic structure of the SiO_x_ and TiO_2_ NPs inserted SiO_x_ system, and density functional theory (DFT) calculations, were conducted with the Vienna Ab Initio Simulation Package (VASP) with MedeA GUI^43,44^. Electronic structures for the α-phase of SiO_2_ and the anatase phase of TiO_2_ were considered to predict the ReRAM switching in the SiO_x_ and TiO_2_ NPs inserted SiO_x_ system. For all calculations, we used the PBEsol functional with 500 eV cut-off energy^45–47^. During unit cell calculation, to have the k-spacing of less than 0.2/Å, we chose a 9 × 9 × 7 and 9 × 9 × 5 grid of k-points for SiO_2_ and TiO_2_, respectively. Geometric optimization was performed using an RMM-DIIS algorithm, iterated until the 0.01 eV/Å condition was satisfied. To determine the electronics structure of defects in SiO_2_ and TiO_2_, we generated 2 × 2 × 1 supercell for both SiO_2_ and TiO_2_. Single oxygen vacancy in various charging states (V_O_^++^, V_O_^+^, V_O_^0^, V_O_^–^, V_O_^–^) were generated in both supercell structures, and geometrically optimized until the 0.01 eV/Å condition was satisfied with a 5 × 5 × 3 grid of k-points and 5 × 5 × 3 grid of k-points for SiO_2_ and TiO_2_, respectively. Calculation with hybrid function (HSE06) was performed to evaluate the accurate position of defect states for optimized structures that contained defects^48^. The formation energy of the charged defect was computed using Eq.^49^:

$$E_{f} = E\left( q \right) - E\left( n \right) + q\left( {\mu_{e} + \Delta V} \right)$$

where E(q) is the total energy of the supercell with charge q, E(n) is the total energy of a neutral supercell, µ_e_ is the chemical potential of electron (Fermi level), and ΔV is the shift of energy level of the valance band maximum. To explain the resistive switching mechanism based on formation/rupture of oxygen vacancies circuit breaker (CB) modeling was conducted using Matlab program. The CB network was connected by horizontal and vertical CB resistors (90 × 30), which were composed with two resistances of R_low_ and R_high_. During the CB modeling, the voltage was applied to all nodes in the top lines, while all nodes in the bottom lines were grounded.

## Data Availability

All data generated or analyzed during this study are included in this published article.
